# Editorials

**Published:** 1891-08

**Authors:** 


### HOMŒOPATHIC PHYSICIAN,

A MONTHLY JOURNAL OF

HOMŒOPATHIC MATERIA MEDICA AND CLINICAL MEDICINE.

"If our school ever gives up the strict inductive method of Hahnemann, we are lost, and deserve only to be mentioned as a caricature in the history of medicine."—CONSTANTINE HERING.

Vol. XI. AUGUST, 1891. No. 8.

### EDITORIALS.

THE I. H. A. MEETING.—He who is in doubt in respect of the efficiency in diseases of all kinds of the law of the similars, and the minimum dose of the potentized drug, should have been present at the Spring House, Richfield Springs, N. Y., during the days of the 23d–26th June ult. He would have met there a band of earnest, conscientious men and women—honest followers of Hahnemann—whose lives are devoted to healing the sick through the instrumentality of that law of nature first elaborated by that wonderful genius, Samuel Hahnemann.

We are sure the doubter would have profited by what was done there—if he desires to learn the truth. We know he could not have gone away without bearing testimony to the honesty of purpose of those who leave their homes and practices and travel, in most instances, hundreds of miles in order to attest by their presence the interest they feel in propagating the truths of genuine Homœopathy.

One of the marked features of these meetings is the enthusiasm of the gray-headed, venerable men who year after year come up with their confirmations of the law, and who vie with the younger men in the fervor with which they attest the truth of the law of the similars.

This is the more marked because it is diametrically opposite to the position taken by old practitioners of old-school medicine, and of that class of false homœopathists who are always attempting to smirch the fair name of Homœopathy. The older the honest follower of Hahnemann grows the more zeal he manifests in his medical work, and he is more fervent in upholding the law. On the other hand, the older the allopath the less ardor he shows regarding the efficacy of drugs in the cure of disease. The mongrel is always in doubt, and, like the more respectable allopath, he flounders about in darkness, without a guide, without a law, and in most instances apparently without a conscience.

It has been, and is now, the part of many of these pseudo-homœopathists to flaut at the honest followers of Hahnemann, to call them visionaries, Hahnemanniacs, and other terms too numerous to mention—but we have never known them to say they are dishonest. To those of this class who desire to know the truth we extend a hearty invitation to attend the next meeting of the International Hahnemannian Association, for we feel convinced that they would then acknowledge that there is more between heaven and earth than was ever dreamt of in their philosophy. To him who despairs of the continuance of genuine Homœopathy, we also extend an invitation, for we are sure his pessimistic ideas will be met by such convincing arguments that he will go away feeling less doubtful of the progress of the good cause.

In 1881, at Coney Island, this Association numbered about twelve members. In 1891 there are nearly two hundred members. Surely there is nothing in this to cause despair!

This last meeting was marked by the character of the papers prepared, and by the discussions. We shall lay before our readers, in this and future numbers, a good part of the work done, and we trust that when another year comes around there will be found on the list of members present with papers, and prepared to impart some of the facts which experience has confirmed, many whose absence was noted at the last meeting.

An innovation was the creation of two new classes of membership—the junior and honorable seniors. To the latter class there were elected Drs. P. P. Wells, Ballard, Seward, and T. P. Wilson. For membership in the former class several applications were received. Junior membership is intended for those who wish to become honest followers of Hahnemann, and who will, after serving as members in this class for a few years, the Board of Censors advising, be elected to active membership, with all the privileges that honor can give. The junior membership idea is due to Dr. Biegler, of Rochester, and we sincerely believe that it will tend to bring into the ranks of the Hahnemannians many who would otherwise never have the opportunity to learn what genuine Homœopathy is capable of doing in treating the sick.

Let us all, then, taking as our examples such workers as Wells, Biegler, Fincke, and the many others whose entire lives are given to advancing the best interests of the sick by devoting themselves to the desire to know and to propagate the best that can be known of Homœopathy, gird up our loins to the work laid out for us by Hahnemann and which has so ably been done by many of his devoted followers. G. H. C.

---

The International Congress and the Institute.—In this number we give a condensed report of the proceedings of the International Congress of Homœopathic Physicians which assembled at Atlantic City, New Jersey, in June. We also add that of the American Institute. The report is taken from the very full account as published in the *Public Ledger* of Philadelphia.

There are thus three important annual conventions of Homœopathy to publish this year. The American Institute, the International Congress, and the International Hahnemannian Association. The notes of the last named have not yet reached us, but we hope to have them in time for our September issue, as the meeting this year was more than usually instructive. See the leading editorial of this number. W. M. J.

---

Dr. Wells on Intermittent Fever.—We must apologize for the absence of the usual quota of Dr. Wells' book on *Intermittent Fever* this month. Our pages are so full that we are obliged to lay aside the work until next month. W. M. J.

